# Supplementary material for: Effects of Insertion of Ag Mid-Layers on Laser Direct Ablation of Transparent Conductive ITO/Ag/ITO Multilayers: Role of Effective Absorption and Focusing of Photothermal Energy
Source: Materials (Basel). 2021 Sep 7;14(18):5136. doi: 10.3390/ma14185136 (PMC8465732; doi:10.3390/ma14185136)
Supplement: Supplementary file 1 [file materials-14-05136-s001.zip › materials-1322507-supplementary.pdf]

materials

Supplementary

Effects of insertion of Ag mid-layers on laser direct ablation of transparent conductive ITO/Ag/ITO multilayers: role of effective absorption and focusing of photothermal energy

Younggon Choi [1,†], Hong-Seok Kim [2,†], Haunmin Lee [3], Wonjoon Choi [3], Sang Jik Kwon [1], Jae-Hee Han [2,*] and Eou-Sik Cho [1,*]

[1] Department of Electronic Engineering, Gachon University, 1342 Seongnam-daero, Sujeong-gu, Seongnam-si 13120, Gyeonggi-do, Korea; cyg1994@gmail.com (Y.C.); sjkwon@gachon.ac.kr (S.J.K.)
[2] Department of Materials Science and Engineering, Gachon University, 1342 Seongnam-daero, Sujeong-gu, Seongnam-si 13120, Gyeonggi-do, Korea; hskim2024@gmail.com
[3] School of Mechanical Engineering, Korea University, 145 Anam-ro, Seongbuk-gu, Seoul 02841, Korea; skekgusals@korea.ac.kr (H.L.); wojchoi@korea.ac.kr (W.C.)
* Correspondence: jhhan388@gachon.ac.kr (J.-H.H.); es.cho@gachon.ac.kr (E.-S.C.)
† These authors contributed equally to this work.

Simulation conditions

1. Material properties

Parameters	Material	Value
Specific heat, C_p ($\mathrm{Jkg^{-1}K^{-1}}$)	Glass	880
	Ag	240
	ITO	753
Density, ρ ($\mathrm{gcm^{-3}}$)	Glass	2.5
	Ag	10.49
	ITO	7.13
Thermal conductivity, κ ($\mathrm{Wm^{-1}K^{-1}}$)	Glass	0.94
	Ag	429
	ITO	11.5
Absorption coefficient, α (m^{-1}) (at 1064 nm)	Glass	50
	Ag	1.03×10^7
	ITO	4×10^5
Reflection coefficient, R (at 1064 nm)	Glass	0.04
	Ag	0.64
	ITO	0.01

2. Laser beam design (Gaussian beam)

$$Q(x,y,z) = Q_0(1-R_c)\frac{A_c}{\pi\sigma_x\sigma_y}e^{-\left[\frac{(x-x_0)^2}{2\sigma_x{}^2}+\frac{(y-y_0)^2}{2\sigma_y{}^2}\right]}\cdot e^{-A_c z}\cdot f(t)$$

(Q_0, total power input; R_c, reflection coefficient; A_c, absorption coefficient; $f(t)$, 10 ns pulse)

3. Specific set parameters of the materials

- Glass substrate: 1 μm
- Ag layer thickness: 6, 13, 16 nm
- Laser beam width: 80 μm
- Laser beam: 1064 nm (wavelength), duration of 10 ns
- Given pulse energy: 0.39 J/cm^2
